# The Usefulness of Mesenchymal Stem Cells beyond the Musculoskeletal System in Horses

**DOI:** 10.3390/ani11040931

**Published:** 2021-03-25

**Authors:** Alina Cequier, Carmen Sanz, Clementina Rodellar, Laura Barrachina

**Affiliations:** 1 Laboratorio de Genética Bioquímica LAGENBIO—Instituto de Investigación Sanitaria de Aragón (IIS)—Instituto Agroalimentario de Aragón (IA2), Universidad de Zaragoza, C/Miguel Servet, 177, 50013 Zaragoza, Spain; alinacs@unizar.es (A.C.); rodellar@unizar.es (C.R.); 2 Servicio de Cirugía y Medicina Equina, Hospital Veterinario, Universidad de Zaragoza, C/Miguel Servet, 177, 50013 Zaragoza, Spain; cap141297@gmail.com

**Keywords:** One Medicine, mesenchymal stem cells, immune-mediated disorders, ophthalmology, reproduction, equine asthma, equine metabolic syndrome, wounds, laminitis, neurologic disorders

## Abstract

**Simple Summary:**

The main target of mesenchymal stem cell therapy in horses has long been the locomotor system, because these athletic animals commonly suffer from tendon and joint lesions. Originally, mesenchymal stem cells were thought to act by just differentiating into the cells of the injured tissue. However, these cells are also able to regulate and stimulate the body’s own repair mechanisms, opening the door to many applications in inflammatory and immune-mediated disorders in both animals and humans. In horses, beyond their traditional application in the musculoskeletal system, these cells have been studied for ophthalmologic pathologies such as corneal ulcers or immune-mediated processes, and for reproductive disorders such as endometritis/endometrosis. Their potential has been explored for equine pathologies very similar to those affecting people, such as asthma, metabolic syndrome, aberrant wound healing, or endotoxemia, as well as for equine-specific pathologies such as laminitis. Current evidence is still preliminary, and further research is needed to clarify different aspects, although research performed so far shows the promising potential of mesenchymal stem cells to treat a wide variety of equine pathologies, some of which are analogous to human disorders. Therefore, advancements in this path will be beneficial for both animals and people.

**Abstract:**

The differentiation ability of mesenchymal stem cells (MSCs) initially raised interest for treating musculoskeletal injuries in horses, but MSC paracrine activity has widened their scope for inflammatory and immune-mediated pathologies in both equine and human medicine. Furthermore, the similar etiopathogenesis of some diseases in both species has advanced the concept of “One Medicine, One Health”. This article reviews the current knowledge on the use of MSCs for equine pathologies beyond the locomotor system, highlighting the value of the horse as translational model. Ophthalmologic and reproductive disorders are among the most studied for MSC application. Equine asthma, equine metabolic syndrome, and endotoxemia have been less explored but offer an interesting scenario for human translation. The use of MSCs in wounds also provides a potential model for humans because of the healing particularities in both species. High-burden equine-specific pathologies such as laminitis have been suggested to benefit from MSC-therapy, and MSC application in challenging disorders such as neurologic conditions has been proposed. The available data are preliminary, however, and require further development to translate results into the clinic. Nevertheless, current evidence indicates a significant potential of equine MSCs to enlarge their range of application, with particular interest in pathologies analogous to human conditions.

## 1. Introduction

The equine industry is a sector of great economic importance, mainly due to the sporting dedication of the horse. Thus, musculoskeletal conditions have a great relevance in these animals. The limited healing capacity of ligaments, tendons, and cartilage have led to the interest in advanced biological therapies [1] aimed at restoring the structure and functions of tissues or organs using the own mechanisms of the body [2], of which mesenchymal stem cells (MSCs) are of particular relevance.

Initially, MSC therapeutics were predicated to be based on their ability to differentiate into cells of the appropriate tissue type, and thus to directly stimulate regeneration of the damaged structures. However, it has been shown that MSCs exert their effects mainly through the secretion of a wide range of bioactive molecules [1], which significantly increases the scope for their therapeutic applications. Actually, the main focus on musculoskeletal application in equine practice is in contrast with human medicine, where MSC therapies are primarily focused on immune-mediated, inflammatory, and ischemic diseases [3].

Even though musculoskeletal pathologies are among the most frequent in horses, the alteration of other systems is also of great relevance provided their economic and welfare impact [4]. The treatment of some of these pathologies presents important limitations owing to different factors, such as horse anatomy and healing physiology in the case of wounds [5], or intrinsic characteristics of immune-based pathologies such as asthma, where only palliative treatments are available [6]. Thus, the range of therapeutic mechanisms elicited by equine MSCs could extend their application beyond the locomotor system. Moreover, numerous naturally occurring diseases in both animals and humans (developmental, infectious, autoimmune, or allergic) have similar pathophysiological bases [7].

This review aims at revising the current knowledge on the use of MSCs for equine pathologies beyond the locomotor system, including ophthalmic and reproductive pathologies, equine metabolic syndrome, equine asthma, wounds, laminitis, neurological disorders, and systemic inflammatory response syndrome, highlighting the value of the horse as a translational model for developing novel treatments that could benefit both animals and humans.

### 1.1. “One Medicine, One Health”: The Role of the Horse

In comparison with small animals such as rodents, large species such as horses better resemble the anatomy and physiology of humans, and their greater lifespan allows for longer-term follow-up. Several organizations such as the European Medicines Agency, the U.S. Food and Drug Administration, and the International Society for Stem Cell Research are recommending the use of large animal models to evaluate the efficacy, durability, dose response, and safety of advanced therapeutic medicinal products [8]. Horses are already recognized as models for several human diseases, including metabolic syndrome [9], asthma [10], musculoskeletal diseases [11,12], melanoma [13], or autoimmune uveitis [14]. Moreover, other equine conditions may also serve as models, including infectious diseases [15], fertility disorders [16], and even depression and mental conditions [17,18].

All of this led to the development of the “One Health, One Medicine” approach, which, according to the World Health Organization, promotes a vision of the health of humans, animals and the environment as a coherent system, and presumes that diseases in humans and animals require analogous therapeutic approaches [19]. Hence, progress in animal models is mutually beneficial for animals, researchers, and human and veterinary patients. Importantly, the rapid advancement of regenerative medicine in the horse makes this species particularly relevant for translational research [20].

### 1.2. Properties of Mesenchymal Stem Cells

Mesenchymal stem cells are adult and multipotent stem cells of mesodermal origin, which have raised interest in the field of regenerative medicine due to their unique biological properties [21]. Equine MSC characterization is based on the recommendations of the International Society for Cell Therapy (ISCT) set for human MSCs, which include cellular plastic adherence, expression of the surface markers cluster of differentiation (CD)90, CD105 and CD73, and the lack of expression of CD45, CD34, CD14 or CD11b, CD79a or CD19 and human leukocyte antigen (HLA) class II. In addition, these cells must be able to differentiate at least into osteoblasts, adipocytes and chondrocytes in vitro [22]. Equine MSCs meet the set criteria of plastic adherence and multipotency, but variably express different surface markers depending on tissue source [23,24].

In equine medicine, MSCs are often the choice for advanced therapies, due to their ease of isolation and culture, their multipotency, and their ability to migrate to damaged tissues [25]. The mechanisms of action through which MSCs exert their effects have not been fully characterized [21]. Initially, it was thought that MSCs differentiated directly into cells of the affected tissue or enhanced the activity of resident cells [3], although it has been reported that MSCs can act indirectly by secreting immunomodulatory and bioactive factors [22,23,24,26,27].

These paracrine effects can be divided into immunomodulatory, anti-scarring, chemoattractant and trophic effects, which can be further subdivided into anti-apoptotic, supportive (stimulation of mitosis, proliferation and differentiation of precursors) and angiogenic. The number of molecules known to mediate the paracrine action of MSCs increases every day [28]; thus indicating that there is a substantial potential to harness these properties to treat several medical conditions in horses.

For example, MSCs secrete different chemokines that recruit and regulate the function of several cell types, as well as growth factors such as vascular endothelial growth factor (VEGF), hepatocyte growth factor (HGF) or the leukemia inhibitory factor (LIF) that promote survival and stimulate the proliferation of resident cells. Some of these growth factors, such as VEGF, also elicit pro-angiogenic effects of great importance, because the restoration of blood supply is essential for the recovery of damaged tissues [25].

In addition, MSCs secrete different cytokines and mediators such as interleukin (IL)-6 and 10, prostaglandin E-2 (PGE-2), transforming growth factor (TGF-β), or nitric oxide (NO), which elicit immunomodulatory actions such as inhibiting the proliferation of T lymphocytes [29], prevent lysis mediated by cytotoxic T cells [30], suppress the activation of natural killer (NK) cells [31] and macrophages [32], or modulate B cell proliferation [33].

## 2. Applications of Equine MSCs in Ophthalmology

The corneal epithelium contains limbal stem cells (LSCs), whose deficiency may greatly affect corneal transparency and integrity of the ocular surface [34]. Mesenchymal stem cells can differentiate into corneal epithelial cells both in vitro and in vivo in rabbits [35]. In addition, MSC paracrine activity can reduce oxidative stress and inhibit the release of pro-inflammatory cytokines, thereby reducing corneal inflammation and neovascularization [36,37]. The in vivo ophthalmologic studies presented here are summarized in Table 1.

### 2.1. Corneal Ulcers and Ulcerative Keratitis

Horses are more prone to corneal damage than other species because of their large-sized eyes placed laterally and prominently [38], and the active movements of their head which favors exposure to bacterial or fungal contamination [39]. Some corneal ulcers can be very severe due to protease activity and can lead to vision loss. The therapeutic strategy should be aimed at eradicating the infection and limiting cornea destruction, in order to control pain and minimize scar formation [40]. Current therapies include topical antibiotics, anti-proteases, and mydriatic or cycloplegic drugs [41], but the typically associated pain makes repeated local administration difficult.

The wound healing capacity of equine autologous bone marrow-derived MSCs (BM-MSCs) and their supernatant (MSC-Sp) was evaluated in vitro using a scratch assay in corneal fibroblasts. The significant decrease in the scratch area after exposure to either MSCs or MSC-Sp suggested their potential to improve corneal healing. Interestingly, the use of MSC-Sp may provide a stem cell-derived but cell-free product that could be more easily stored and applied [42].

Regarding in vivo studies, in one case of bacterial ulcerative keratitis unresponsive to conventional therapies, a single dose of autologous peripheral blood-derived stem cells (PB-SCs) was applied both systemically (intravenously (IV), jugular vein) and in transverse facial artery. In addition, local application was performed three times a day for seven days. Three months later, the ulcer was almost unnoticed and the clinical signs of inflammation, pain and irritation disappeared [43]. Similar results were observed in another case of bacterial ulcerative keratitis and in three cases of corneal ulcers treated with IV and local administration of autologous PB-SCs [40].

### 2.2. Equine Recurrent Uveitis

Equine recurrent uveitis (ERU) is a spontaneous and immune-mediated disorder characterized by recurrent episodes of intraocular inflammation separated by periods of remission [44]. The exact pathophysiology of ERU is not clear, although it is thought that *Leptospira interrogans* can be implicated by initiating an infection that leads to ocular immune privilege breakdown [45]. The subsequent immune response involves cytokines and chemokines that activate helper T cells (Th), and Th17-associated cytokines seem to play a role [46]. Currently, there is no cure, and treatment is focused on preserving vision, alleviating pain, and limiting the recurrence of episodes by using mydriatics and anti-inflammatory drugs. The end stage of the disease in the majority of affected horses is blindness [44].

Mesenchymal stem cells are effective at reducing immune cell activation in vitro in many species, making them a potential therapeutic option for ERU [47]. It has been shown in several mammals, such as cats, dogs and horses, that MSCs can induce a switch from pro-inflammatory to regulatory T cell subsets when applied in immune diseases [47,48,49]. The same has been suggested for ERU in an in vitro assay in which lymphocytes from ERU-affected horses were co-cultured with adipose-derived MSCs (AT-MSCs) [50]. However, to the best of the authors’ knowledge, there is no literature on controlled equine clinical studies employing MSCs in ERU except for a brief mention in the study of Saldinger et al., which stated satisfactory treatment of three cases (unpublished data).

Interestingly, ERU closely resembles human autoimmune uveitis regarding clinical and immune pathological features, including the same autoantigens involved and the remitting-relapsing onset of the disease. Therefore, ERU has been suggested as a reliable spontaneous model to study the histopathological changes and the inflammatory processes in uveitis [14,51]; thus, therapeutic advancements in ERU and human uveitis can be mutually beneficial.

### 2.3. Equine Immune Mediated Keratitis

Equine immune mediated keratitis (IMMK) is a generic term used to describe a heterogeneous group of chronic, non-ulcerative corneal opacities accompanied by intraocular inflammation. The etiopathogenesis is unknown, but seems to be related with dysregulated immune responses involving a complex cytokine cascade and amplified pro-inflammatory response [41]. Long-term topical anti-inflammatory and immunosuppressive therapy is the main management protocol, although it does not offer a definitive solution and can be challenging to maintain with low owner compliance or poor response to treatment [52].

Due to the immunomodulatory action of MSCs, these have been suggested as potential therapeutic tools. One case of IMMK was reported to have a positive response after two intravenous injections of autologous PB-SCs and local instillation, without signs of relapse [40]. In another study involving four horses with IMMK unresponsive to conventional treatment, subconjunctival injection of autologous BM-MSCs (1–5 injections every 3–4 weeks) was tested along with usual treatment. Three horses had a positive clinical response, as demonstrated by decreased corneal opacity, diminished neovascularization, and improvement in surface irregularity [53].

The limitations of most in vivo ophthalmologic studies include a small sample and variability in patient selection, along with lack of a control group. Nevertheless, all cases were poorly responsive to medical management and showed improvement after MSC therapy. The administration protocol, including route, number of applications, and duration of the treatment, as well as medical management before and during MSC therapy, varied significantly. Regarding the route of administration, the subconjunctival space is relatively common for ocular medications and provides high drug concentration to the cornea and anterior structures for prolonged periods [54]; therefore, it could represent a suitable approach for local MSC administration that avoids repeated manipulation of the painful eye for local instillation, which also poses the problem of prolonged storage [43]. Other delivery systems include local intra-arterial injection in order to reach the internal structures of the eye, and contact lenses seeded with MSCs have been used in other models [55]. Further research based on controlled studies and standardized protocols supported by distribution assays is needed to demonstrate the benefits of such treatments and implement its use in clinical practice.

## 3. Applications of Equine MSCs in Reproduction

Due to the MSC regenerative and immunomodulatory properties, these might be used to treat damaged reproductive tissues, as has been proposed in different animal models, such as rats [56]. Table 2 summarizes the in vivo reproduction-related studies discussed below.

### 3.1. Endometritis

Endometritis is the infection and/or inflammation of the endometrium, and constitutes the main cause of subfertility and is the third most common disease affecting horses [57]. Repeated inflammation of the endometrium can lead to endometrosis, a chronic state characterized by fibrosis with glandular alterations [58]. Both persistent breeding-induced endometritis (PBIE) and endometrosis are of pivotal importance for reproductive health in mares.

#### 3.1.1. Equine Persistent Breeding-Induced Endometritis

Persistent breeding-induced endometritis is an acute inflammatory response of the endometrium to semen, linked to an incapability of the uterus to remove bacteria, spermatozoa, and inflammatory exudate post-breeding. This disease affects broodmares of all breeds, leading to reproductive inefficiency and important economic losses [59]. Traditional therapeutic modalities lack sufficient effectivity, which along with increasing antibiotic-resistances, has led to the development of alternative therapies such as MSCs [60], because of their engraftment and immunomodulatory abilities.

Studies in healthy mares have provided a first insight into the effects of MSCs on reproductive tissues. Autologous MSCs isolated from endometrial biopsies harvested from healthy mares were infused into both uterine horns during the early diestrus to avoid ready elimination from the uterine lumen. Endometrial mesenchymal stem cells (eMSCs) were detected in the uterine lumen up to 24 h after infusion, but they did not engraft into the endometrium. Moreover, eMSCs effectively attenuated the inflammatory response produced by the uterine infusion itself [61]. Another study showed that allogenic BM-MSCs infused into the uterus 24 h before insemination modulated the uterine inflammatory response in healthy mares [62]. Subsequently, PBIE was induced in nine healthy mares which received intrauterine instillation of allogeneic AT-MSCs or eMSCs. The MSC administration significantly reduced inflammation regardless of the origin of the cells, but their engraftment after one month was limited, suggesting that their function in this context is preferentially exerted by paracrine mechanisms [63].

#### 3.1.2. Endometrosis

Endometrosis is characterized by periglandular and/or stromal endometrial fibrosis, including glandular alterations within fibrotic foci. The etiology is not fully elucidated, but it seems to be age-related [64]. The endometrial changes caused by this disease are irreversible, and therapeutic management is challenging [65].

In an ex vivo model, endometrial biopsies from healthy and pathological mares were exposed to allogeneic AT-MSCs, which were able to infiltrate both the periglandular space and single glands of endometrosis-affected tissue [66]. In an in vivo study, autologous BM-MSCs were injected directly into the uterus using a catheter in mares with subfertility history and degenerated endometrium to assess safety in pathological conditions, observing no clinical alterations, histological changes, or endometrial edema [67]. These studies suggest the feasibility and safety of endometrial injection of MSCs as a new therapeutic approach for uterine disorders.

Another study reported that allogeneic AT-MSCs infused in the uterine lumen of six endometrosis-affected mares effectively homed into both the glandular and non-glandular endometrium when delivered by a simple technique, similar to that used for artificial insemination [68]. A continuation of this study showed histological improvement of endometrosis after infusing allogeneic AT-MSCs. The authors suggested that multiple mechanisms, including homing to fibrotic areas and increased epithelial cell proliferation, might mediate the anti-scarring effects observed [69]. Furthermore, amniotic membrane-derived MSCs may improve the endometrial cell replenishment when scarcity or low proliferation of endometrial cells is associated with pregnancy failure [70].

Compared to BM-MSCs, eMSCs have a higher ability to differentiate into smooth muscle [71], and display robust immunomodulatory [72] and migratory properties [73]. Moreover, eMSCs can be collected from simple endometrial biopsies, which can be safely and repeatedly obtained, thus suggesting a novel source of therapeutic cells for inflammatory conditions of the uterus [74].

### 3.2. Ovarian Diseases

Intra-ovarian injection of allogeneic BM-MSCs to treat ovarian dysfunction in old mares has been suggested in one study. Although there were no side effects, MSC injection was not associated with significant changes in follicle number, oocyte recovery and maturation rate, or blastocyst rate [75]. This contrasts with MSC beneficial effects observed in chemotherapy-damaged ovaries in other species [76,77], but these data should be extended to be conclusive, because to the best of the authors’ knowledge, there are no further studies exploring this possibility.

### 3.3. Testicular Diseases

Testicular disorders are characterized by altered or suppressed activity leading to important reproductive issues in stallions and consequent economic impacts [78]. Immunomodulatory, anti-inflammatory, and anti-apoptotic effects of MSCs, as well as their ability to migrate to injured tissues, support their potential to treat testicular disorders, as suggested in an induced testicular torsion model in rats [79]. One in vivo study evaluated the intratesticular injection of allogeneic BM-MSCs in healthy stallions. The absence of clinical abnormalities and altered semen parameters suggested that this is a safe procedure [80], although further studies are required to test their therapeutic potential.

## 4. Application of Equine MSCs in Metabolic Disorders

### Equine Metabolic Syndrome

Equine metabolic syndrome (EMS) is an endocrine disorder characterized by pathological obesity, insulin dysregulation, altered hepatic function, and predisposition to developing laminitis. Obesity is the main risk factor for EMS, because adipose tissue acts as an important secretory organ producing different molecules such as pro-inflammatory cytokines with associated adverse local and systemic effects [81]. Management of EMS should include a well-balanced diet and physical activity. Some drugs such as metformin can help regulate this disorder, although there are no treatments available that can effectively resolve the EMS [82].

Therefore, MSCs have been proposed as a therapeutic strategy in different metabolic syndromes in several species, such as rodents [83,84,85], dogs [86] and monkeys [87]. Importantly, great attention has been paid to MSC treatment of human diabetes type 2 [88,89]. Thus, the One Medicine concept applies in both directions; knowledge from human studies is also highly valuable for veterinary medicine.

Adipose-derived MSCs from EMS-affected horses display senescent phenotype, increased apoptosis, and reduced viability and differentiation capacity, which would make autologous therapy suboptimal [90,91,92]. In a recent study of the same group, autologous AT-MSCs were exposed in vitro to pharmacotherapy with 5-azacytidine (AZA) and resveratrol (RES) before their clinical application in order to reverse the aged phenotype of these cells. These rejuvenated autologous AT-MSCs showed in vivo potential to improve liver metabolism in one EMS-diagnosed horse, as demonstrated by a decrease in specific liver enzymes. Nonetheless, MSC therapy was combined with conventional EMS management, which could have also contributed to this improvement [93].

## 5. Application of Equine MSCs in the Respiratory System

### Equine Asthma

Equine asthma syndrome encompasses a spectrum of inflammatory airway diseases characterized by chronic respiratory signs ranging in severity that can significantly affect athletic performance [6]. Although its exact etiopathogenesis remains incompletely defined, immune-mediated responses are involved and lead to excessive mucus production, neutrophilic accumulation, bronchial hyperreactivity, and bronchospasm [94]. Treatment is limited to environmental management, anti-inflammatory drugs, and bronchodilator therapy. Nevertheless, the use of inhaled corticosteroids may be contraindicated in some cases, and may also be cost-prohibitive and inconvenient for some owners [6].

Studies in murine models of induced asthma have shown that MSCs may be beneficial for managing this process [32,95,96]. To date, there are no published reports on the use of MSCs in vivo to treat equine asthma. However, the use of cell derivatives has been tested. An in vitro assay showed the ability of conditioned medium and microvesicles from equine amniotic mesenchymal cells to modulate lipopolysaccharide (LPS)-stimulated equine alveolar macrophages, suggesting that these products can play a role in treating inflammatory diseases of the lung [97]. In vivo, the intra-tracheal instillation of autologous bone marrow-derived mononuclear cells in eight asthma-affected horses showed that the procedure is safe and improved clinical signs, suggesting amelioration of the asthmatic inflammatory response [98].

Moreover, equine and human asthma syndromes share several features, with the difference that the process is dominated by neutrophils in horses and by eosinophils in humans [6]. The similarities in the airway remodeling processes make equine asthma an ideal model to study the cellular and molecular pathways associated with the asthmatic airway response and its reversibility [10].

## 6. Application of Equine MSCs in Disorders of the Integumentary System

The main findings of in vivo studies using MSCs for integumentary-related disorders are highlighted in Table 3.

### 6.1. Wounds

Due to their “flight” instinct in response to frights, horses are particularly susceptible to trauma [99]. Actually, wound injuries are the most common medical condition affecting horses [100,101]. Traumatic wounds commonly occur in the distal limb, where healing is often delayed due to high skin tension, minimal vascularization, and a low-grade inflammatory response. This aberrant inflammation leads to a fibroproliferative response, resulting in a dysplastic healing with exuberant granulation tissue [102]. These particularities make successful wound management difficult, to which MSCs could contribute owing to their varied paracrine activities.

In vitro, equine MSCs promote dermal wound repair through the mobilization of dermal fibroblasts and by increasing the expression of genes involved in wound healing [103]. In an equine distal limb wound model using six horses, allogeneic umbilical cord blood-derived MSCs (UCB-MSCs) were applied after culture in normoxic or hypoxic conditions, either directly injected into wound margins or topically applied embedded in an autologous fibrin gel. Early healing was enhanced, with histology suggesting a pro-healing rather than a pro-inflammatory scenario and smaller sizes in MSC-treated wounds, with no additional advantage of hypoxic preconditioning [104]. Systemic administration of allogeneic UCB-MSCs resulted in engraftment into induced wounds on the forelimb and thorax at early stages, without clinically adverse reactions [105]. Autologous PB-SCs injected both locally and systemically in four naturally occurring chronic dermal wounds in the metatarsus, unresponsive to conventional treatments, showed positive outcomes with granulation tissue formed within four weeks, and no adverse effects were noted [106]. These results suggest MSCs as a promising tool to improve and accelerate the healing of chronic wounds.

Equine distal limb wounds display similarities to human wounds, with keloid formation in the latter being similar to exuberant granulation tissue formation in horses. Humans and equines are the only two species that spontaneously develop these fibroproliferative disorders, making the horse a suitable model for studying the pathogenesis and treatment of keloids and hypertrophic scars [99]. In both species, chronic wound management is a growing problem leading to a significant economic impact; thus, there are demands for advanced therapeutic options able to decrease healing time and minimize complications.

### 6.2. Decubitus Ulcers

Decubitus ulcers result from pressure, shear, and/or friction when the patient is lifted or put in decubitus, leading to tissue ischemia, cell death and necrosis [107]. Thus, the pathogenesis of this injury differs from that in traumatic wounds. Neonatal foals are prone to decubitus ulcers due to their thin skin and possible concomitant disorders, which force them into prolonged decubitus [108]. Mesenchymal stem cells play a key role in skin homeostasis and repair by promoting cell differentiation, immunomodulation, and the secretion of growth factors to drive re-epithelialization and neovascularization [109].

In a septic neonatal foal, local implantation of amniotic fluid-derived MSCs (AF-MSCs) along with platelet-rich plasma (PRP) gel effectively improved decubital ulcers earlier than in those ulcers treated with *Aloe vera* gel only [110]. In another study, several deep sore wounds, presented concomitantly in five septic foals, were treated by either allogeneic AF-MSCs embedded in a carboxymethylcellulose scaffold or by a commercial preparation of formosulfathiazole, the former leading to faster healing [111]. Additionally, the effectiveness of local application of allogeneic Wharton’s jelly MSCs (WJ-MSCS) was reported in a six-month-old filly with a large non-healing skin wound in the hock [112]. Therefore, MSCs may be considered for healing skin ulcers in foals, with no side effects noticed, although data are limited to a few reports including low numbers of animals.

### 6.3. Laminitis

Laminitis is included as part of the integumentary system provided the distal third phalanx is enveloped by a specialized epithelial tissue, the corium, attaching the bone to the hoof. However, it should be noted that this is a complex and multifactorial disease [113]. This disorder affecting the hooves of ungulates is of particular importance in equids, carrying a poor prognosis, with a strong economic impact and constituting a serious issue for animal welfare that may eventually require euthanasia [114]. A great variety of initiating causes can lead to the onset of the disease and can be influenced by different body systems, including inflammation, metabolic disorders, and endothelial or vascular dysfunction [113]. Whatever the triggering factors are, the release of pro-inflammatory mediators and the activation of metalloproteinases leads to the degradation of the basement membrane that may result in a complete mechanical collapse of the foot [115].

The largely incomplete knowledge of the pathogenic mechanisms involved in laminitis makes both prevention and treatment difficult [116]. Laminitis has been associated with the loss of p63+ epidermal stem cells in the hoof lamellae, suggesting limited proliferative potential of the laminitic hoof epithelium [117]. Therapy with MSCs is a promising tool to improve cell proliferation and tissue quality, as well as contribute to vascular stabilization and control the pro-inflammatory environment.

In clinical studies on chronic refractory laminitis, allogeneic UCB-MSCs were delivered directly to the affected foot via regional perfusion (digital vein). The outcome suggested a positive MSC effect on the prognosis of animals treated early, as reflected by the evolution with decreased radiologic distance between the bone and hoof wall [114,118]. Allogeneic BM-MSCs provided better results when digitally perfused compared to epidermally injected [119]. Another study on chronic laminitis administered allogeneic and autologous AT-MSCs suspended in autologous PRP by distal digital venous injection. Improvement of both hoof quality and animal mobility was reported, improving the life quality of treated horses [120].

These results encourage further exploring the use of MSCs to treat chronic laminitis, but it is important to highlight that MSC administration does not eliminate the need for routine management and appropriate hoof support.

## 7. Application of Equine MSCs in Neurological Disorders

Neurological disorders affecting the brain and spinal cord can represent a therapeutic challenge, and many horses can have sequelae even after recommended treatment [121]. The neuroprotective effects of MSCs have been described in other species and involve anti-inflammatory, immunomodulatory, pro-angiogenic and trophic mechanisms [122,123], which could ameliorate the symptoms of several neurodegenerative disorders [124]. Furthermore, MSCs can trans-differentiate in vitro into neuronal lineages [125]. In vivo related studies in horses are summarized in Table 4.

### 7.1. Peripheral Nerve Injury

Horses suffer injury to peripheral nerves from trauma, metabolic and genetic disorders, toxins or degenerative and infectious diseases [126]. The degree of restoration of nerve function depends on the severity and chronicity of the damage, with the worst prognosis in cases where the nerve is transected [127]. There are few surgical techniques for repairing nerves, and clinical results are often poor [128]. Consequences frequently include poor performance, disability, or even death [129,130].

In an in vivo model of acute peripheral nerve injury using three horses, allogeneic BM-MSCs were implanted into the fascia surrounding the *ramus communicans* of one forelimb after a portion was transected. No evidence of nerve regeneration was observed, neither were histological differences between MSC-treated and control injuries found 45 days later [127]. Nevertheless, improvement of nerve regeneration after MSC treatment has been observed in other large animals such as sheep [131].

### 7.2. Wobblers Syndrome

Wobblers syndrome, also known as cervical vertebral stenotic myelopathy (CVSM) or incoordination syndrome, is characterized by ataxia and weakness, caused by the narrowing of the cervical vertebral canal and/or compression of the spinal cord. Medical treatment commonly includes steroidal and nonsteroidal anti-inflammatory drugs, along with diet and exercise restrictions. Surgery can be considered in some cases, but this option is expensive and involves significant risks [132].

An intrathecal injection could extensively deliver cells through the cerebrospinal fluid to reach the equine central nervous system. To evaluate the feasibility and safety of intrathecal transplantation of cells, autologous BM-MSCs were administered to healthy horses and no clinical alterations were developed [133]. A posterior study aimed at determining the safety of a relatively high dose of intrathecal allogeneic AT-MSCs in both healthy and CVSM-affected horses. Neurological status was not altered regardless of atlanto-occipital or lumbosacral administration. Atlanto-occipital injection is apparently distributed more efficiently through the subarachnoid space, suggesting that this approach might be more suitable for cranial spinal cord lesions. As for diseased horses, MSCs could not be found at 15 days after injection at the site of injury, so either cells did not reach the lesion site or did not persist at that time [134].

### 7.3. Laryngeal Hemiplegia or Left Recurrent Laryngeal Neuropathy

Recurrent laryngeal neuropathy is characterized by varying degrees of arytenoid paralysis, constituting a highly prevalent pathology of the upper airway in horses [135]. Affected horses emit abnormal respiratory sounds and may present exercise intolerance in severe cases. Although the overall success rate of laryngoplasty with or without ventriculo-cordectomy is elevated, post-operative complications such as a gradual loss of abduction are very common [136].

To explore the feasibility of using MSCs to promote nerve function restoration, a nerve-stimulator-guided injection of muscle-derived autologous MSCs near the left recurrent laryngeal nerve was performed in five healthy horses. Laryngeal function was not affected, thus suggesting that this delivery technique is safe [137]. These findings would facilitate future studies assessing MSC effectiveness to treat this pathology, which has not yet been tested, to the best of the authors’ knowledge.

## 8. Application of Equine MSCs in Endotoxemia

Systemic inflammatory response syndrome (SIRS) is characterized by an exaggerated inflammatory response to an aggression, which causes a series of unspecific clinical signs that can seriously compromise the patient’s life. This process is of great importance in horses due to the particularities of their inflammatory response and the high incidence of pathologies such as acute abdominal syndrome, pneumonia, and metritis, which can provoke an endotoxemia or sepsis and subsequent SIRS development. These processes can lead to complications such as disseminated intravascular coagulation, vascular endothelial damage, laminitis, and multiple organ dysfunction syndrome [138]. Therefore, SIRS is associated with a significantly higher risk of death in horses presenting acute colic [139] or other disorders. There are no specific treatments for endotoxemia and SIRS other than controlling the primary cause and providing supportive therapy, mostly based on fluids and antiendotoxics [138]. The complexity and poor prognoses of these processes and the lack of effective treatments have led to the interest in using MSCs because of their immunomodulatory capacity. Actually, there are a number of studies in rodent models of endotoxemia showing promising results of the administration of MSCs, including decreased levels of circulating proinflammatory cytokines and increased survival rates [140].

In horses, and to the best of the authors’ knowledge, there is only one study reporting the effects of administering MSCs in an experimental model of endotoxemia [141]. In this study, lipopolysaccharide was IV injected into six horses, and three of them immediately received 100 × 10^6^ allogeneic BM-MSCs IV. Serial clinicopathological assessment and determination of pro-inflammatory cytokine production did not show significant differences between treated and control animals, but adverse reactions after MSC infusion were not observed either, thus suggesting that the procedure is safe; however, further studies with higher numbers of animals and studying variables such as the dosage and moment of administration are needed to elucidate the potential benefit of this therapy. Importantly, endotoxemia, sepsis, and SIRS also affect a high percentage of human patients in intensive care units, and the horse has been proposed as a model for understanding human innate immunity and shedding light onto pathology processes and therapeutics [142].

## 9. Conclusions and Future Perspectives

The unique properties of MSCs hold a great potential for the treatment of different equine diseases other than musculoskeletal injuries, for which current pharmacologic or surgical approaches often do not provide satisfactory results. 

Several studies have explored the use of MSCs for different equine diseases beyond the locomotor system. Their application for ophthalmologic and reproductive disorders has been particularly investigated, and the treatment of wounds, asthma, and SIRS holds a special interest to develop One Medicine approaches. However, the variability among clinical case conditions, source of the MSCs used, cell isolation and culture techniques, and therapeutic protocols (MSC dose, route, number, and frequency of administrations), as well as the low sample size and lack of control groups in some of the studies, limit extracting definitive conclusions. Nevertheless, case reports are highly valuable as a proof of concept, indicating that there is potential for in further investigations. In addition, in vitro studies provide interesting preliminary insight into MSC mechanisms for each pathology, and in vivo studies in healthy animals enable safety and feasibility assessments.

More in-depth research is needed to test the safety and efficacy of these novel treatments, and future clinical trials would include a larger number of similar cases and standardized measurements of the outcomes, in order to establish specific therapeutic protocols. Furthermore, more in vitro and experimental work is needed to understand the pathways through which MSCs elicit their effects, in order to achieve their highest therapeutic potential. Therefore, researchers and clinicians should work together to develop evidence-based treatments and exploit the MSC potential by extending their use to different pathologies in both equine and human patients.

## Figures and Tables

**Table 1:** Ophthalmology in vivo studies using stem cells in horses. Peripheral blood stem cells (PB-SCs), phosphate-buffered saline (PBS), equine recurrent uveitis (ERU), bone marrow-derived mesenchymal stem cells (BM-MSCs), immune-mediated keratitis (IMMK), intravenous (IV).

Pathology	Study	Type of MSCs	Experimental Design	Administration Regime	Outcome	Considerations
Corneal ulcers	Spaas et al., 2011 [43]	Autologous PB-SCs	Naturally occurring pathology (one case): 20-year-old gelding, with bacterial (*Pseudomonas aeruginosa*) ulcerative keratitis, resistant for 6 months against conventional therapies and surgical intervention.	A one-time injection in the jugular vein (125 × 10^3^ PB-SCs in 5 mL PBS) and in transverse facial artery (125 × 10^3^ PB-MSCs in 5 mL PBS) + local application (eye drops formulation, 500 × 10^3^ PB-SCs in 5 mL PBS) 3 times/day, 7 days.	2 weeks: inflammation and tear flow decreased; ulcer size reduced 3 months: eye ulcer almost invisible and inflammation, pain and irritation disappeared. Better general condition.	Single case with no control Cells used not expanded and not fully characterized as MSCs After 7 days, eye drop application was stopped because the cell suspension appeared cloudy, probably because of cell death. Storage conditions of drop bottle between administrations not specified.
	Marfe et al., 2012 [40]	Autologous PB-SCs	Naturally occurring pathology (4 cases) Case 1: 20-year-old gelding with bacterial (*Pseudomonas aeruginosa*) ulcerative keratitis resistant for 6 months against conventional therapies and surgical intervention Case 2: 7-year-old mare with a corneal ulcer treated for 2 weeks and IMKK treated for a year Case 3: 12-year-old gelding with traumatic corneal ulcer treated for 6 months Case 4: 13-year-old gelding with ERU-derived corneal ulcer treated for 2 weeks	1–2 systemic administration (IV) + local instillation 2–3/day for 2 weeks.	Case 1: 2 weeks: inflammation and lacrimation decreased; ulcer size reduced and stable. 3 months: eye ulcer reduced, inflammation stable, pain and irritation disappeared. >3 months: eye ulcer disappeared Case 2: 1 month: no signs of relapse Case 3: 2 weeks: ulcer significantly reduced 1 month: deposit of melanin (scarring effects) Case 4: 2 months: corneal ulcer completely disappeared	Low number of cases and with corneal ulcers of different origins Absence of control group, but animals unresponsive to previous treatments Cells used not expanded and not fully characterized as MSCs Number of PB-SCs administrated not stated Administration route and protocol unclear at some points
IMMK	Marfe et al., 2012 [40]	Autologous PB-SCs	Naturally occurring pathology (one case): 7-year-old mare, poorly responsive to traditional medical treatments for a year (Case 2 above)	2 systemic administration (IV) + local instillation 2/day for 2 weeks	1 month: no signs of relapse 3 months: complete recovery of clinical signs	A single case with no control group Number of PB-SCs administered not stated
	Davis et al., 2019 [53]	Autologous BM-MSCs	Naturally occurring pathology (4 cases): unilateral IMMK poorly responsive to traditional medical treatments Case 1: 9-year-old gelding with midstromal keratitis Case 2: 12year-old mare with superficial to midstromal keratitis Case 3: 9-year-old gelding with midstromal keratitis Case 4: 10-year-old gelding with midstromal keratitis	Subconjunctival injection (15 × 10^6^ MSCs in 1 mL PBS) every 3–4 weeks for 1–5 injections Case 1: 1 single injection Case 2: 3 injections every 3–40 weeks Case 3: 3 injections every 3–6 weeks Case 4: 3 injections every 3 weeks Cases 2 and 3 received 1 and 2 additional injections, respectively	3 weeks: improvement of clinical signs (decreased fibrosis/opacity, irregularity, and vascularization). No relapse for average 1 year. Case 1, 2 and 3: Resolution of fibrosis and neovascularization Case 4: Enucleation due to disease worsening and discomfort	Low number of cases with no control group, but selected upon disease similarities and unresponsiveness to medical treatment. Variability in the administration protocol Additional topical treatments in some case in conjunction with the BM-MSCs (cyclosporine, bromfenac, diclofenac or flunixin meglumine)

**Table 2:** In vivo studies using mesenchymal stem cells in equine reproduction. Endometrial mesenchymal stromal cells (eMSCs), phosphate-buffered saline (PBS), polymorphonuclear neutrophils (PMNs), bone marrow mesenchymal stem cells (BM-MSCs), anti-Müllerian hormone (AMH), autologous conditioned serum (ACS), lactate ringer (LR), intrauterine (IU), post-breeding induced endometritis (PBIE), adipose tissue MSCs (AT-MSCs), artificial insemination (AI), fetal bovine serum (FBS), dimethyl sulfoxide (DMSO), interleukin 6 (IL-6), tumor necrosis factor alpha (TNFα), intracytoplasmic sperm injection (ICSI).

Pathology	Study	Type of MSCs	Experimental Design	Administration Regime	Outcome	Considerations
Safety and distribution assays in healthy mares	Rink et al., 2018 [61]	Autologous eMSCs	Intra-uterine administration of labelled eMSCs and follow-up: Uterine cytology Tracking of eMSCs in peripheral blood Tracking of eMSCs in uterus (lumen and endometrial biopsies) 7 healthy cycling mares, 4–7 years old.Four of these mares were used as controls, two estrous cycles before the eMSC infusion cycle.	15 × 10^6^ eMSCs in 1 mL or PBS infused into each uterine horn during early diestrus (day 4 after ovulation).	Mild inflammatory reaction after infusion was attenuated by eMSCs (percentage of PMNs lower in eMSC than PBS-infused mares at 6h) eMSCs detected in the uterine horn lumen for up to 24 h after infusion but did not migrate into healthy endometrium. eMSCs were not found in the peripheral blood at 6, 12, and 24 h after application.	eMSCs intra-uterine administration is safe and cells persist in the uterine lumen for up to 24 h after infusion, but do not engraft into healthy endometrium at that time.
PBIE	Ferris et al., 2014 [62]	Allogeneic BM-MSCs	Evaluate the ability of ACS, BM-MSCs or dexamethasone to modulate the inflammatory response to spermatozoa after breeding (24 h) 12 healthy mares Experiment 1: Crossover study Experiment 2: Two-way crossover study Administration of treatments, sperm challenge and follow-up: Ultrasonographic evaluations Lavages: PMN and cytokine analysis	Experiment 1: 6 mares treated with an IU infusion of: 20 mL of ACS in 20 mL PBS 20 mg of dexamethasone QS in 20 mL PBS Control: 20 mL PBS One treatment per estrous in the same mares (every 3 weeks). Experiment 2: 6 mares treated with an IU infusion of: 20 × 10^6^ allogeneic BM-MSCs in 20 mL LR 20 mL PBS One treatment per estrous in the same mares (every 3 weeks).	BM-MSC and ACS were able to modulate the uterine inflammatory response to spermatozoa in normal mares Decreased neutrophil migration into the uterine lumen in response to insemination after BM-MSC treatment may be due to increased anti-inflammatory cytokine IL-1Ra and reduced proinflammatory mediator IL-1	Healthy mares (proof of concept for PBIE-affected mares). Same mares used for different treatments. Age of mares not stated.
PBIE	Navarrete et al., 2020 [63]	Allogeneic AT-MSCs and eMSCs	Evaluate anti-inflammatory and engraftment properties of AT-MSCs and eMSCs from the same donors in vivo in mares with induced PBIE. 9 healthy mares with induced PBIE. Follow-up: Lavages: cytokine and gene expression analysis Uterine biopsies	2 × 10^7^ AT-MSCs (*n* = 3) or eMSCs (*n* = 3) in 20 mL of NaCl 0.9% Control group (*n* = 3): 20 mL NaCl 0.9%	Both MSC types significantly reduced inflammation and showed limited engraftment, detectable after one month of infusion Decrease in IL-6 and TNFα in both MSC-treated groups over control.	Age of mares not stated. Virgin mares with no previously reported PBIE (possibility of natural PBIE resistance)
Endometrosis	Alvarenga et al., 2016 [67]	Autologous BM-MSCs	Evaluate the feasibility and safety of MSC endometrial injections 16 mares (15–24 years) with reproductive history of subfertility and endometrial degeneration Control: baseline values before endometrial injection Follow-up: Ultrasonographic evaluations Uterine biopsies	12 endometrial injections of 1 × 10^6^ MSCs in 0.5 mL PBS, each one at 12 different sites, 1 cm apart from one uterine horn to another (12 × 10^6^ MSCs in total)	Neither clinical alteration nor intrauterine fluid and endometrial edema were observed after MSC administration. No histological worsening The results suggest that the procedure is safe	Proof of concept for safety. Therapeutic effects not thoroughly assessed.
	Mambelli et al., 2013 [68] Mambelli et al., 2014 [69]	Allogeneic AT-MSCs	Evaluate the feasibility of an MSC delivery system for endometrosis-affected mares. 6 mares (6–21 years) with varying degrees of naturally occuring endometrosis Follow-up: Uterine biopsies 7, 21 and 60 days	20 × 10^6^ AT-MSCs in 20 mL NaCl 0.9% inoculated into uterus using a technique similar to AI Control (*n* = 2): 20 mL NaCl 0.9%	Migration of AT-MSCs to the uterine body and both horns. Engraftment in both glandular and periglandular spaces in three mares. AT-MSCs beneficially modulated the expression pattern of secretory proteins and promoted proliferation of glandular epithelial cells.	Small control group Different degrees of endometrosis among recruited mares
Ovarian and testicular diseases	Grady et al., 2019 [75]	Allogeneic BM-MSCs	Determine if intra-ovarian injection of BM-MSCs improves or restores ovarian function in aged mares 8 aged mares (20–29 years old) and 6 young mares (7–12 years old) Assessment (aged and young mares): Antral follicle count Serum AMH Assessment (aged mares only): Oocyte meiotic and developmental competence Gross and histological ovarian assessment Gene expression in ovarian tissue as assessed by RNA sequencing.	2 intraovarian injections of 10 × 10^6^ BM-MSCs (different donors) in 1mL (95%FBS and 5%DMSO) in four different locations per ovary Aged mares: First injection Group 1 (*n* = 3): BM-MSCs from donor A Group 2 (*n* = 3): BM-MSCs from donor B Group 3: Control (*n* = 2): 95%FBS and 5%DMSO Second injection: at 6 weeks from different donor Young mares: Group 1 (*n* = 2): BM-MSCs from donor A Group 2 (*n* = 2): BM-MSCs from donor B Group 3 (*n* = 2): Control (95% FBS and 5% DMSO)	No adverse events after intra-ovarian injections were observed Oocyte recovery on follicle aspiration, oocyte maturation, and blastocyst development rates after ICSI remained unchanged in the MSC-treated aged mares, suggesting no detriment of ovarian function but neither a beneficial effect. Injection of BM-MSCs not associated with significant changes in follicle number in young mares. No significant changes in peripheral AMH concentrations in aged and young mares observed, indicating a lack of effect on growing follicles.	BM-MSCs administered immediately after thawing (viability not stated) Small size of groups
	Papa et al., 2020 [80]	Allogeneic BM-MSCs	Evaluate the effect of intratesticular injection of BM-MSCs in healthy stallions, and its outcome on seminal parameters and fertility Experiment 1: 24 stallions (3–4 years) Experiment 2: 3 stallions (3–10 years) Assessment: Experiment 1: Testicular morphometry, thermography Testosterone concentrations Ultrasonography Histopathology (after orchiectomy) Experiment 2: Sperm collection: concentration, kinetics, plasma membrane integrity Insemination of six healthy mares (8 ± 3.5 years old)	Experiment 1: Intratesticular injection 10 × 10^6^ BM-MSCs in PBS in 5 different points (1 mL in each point) (*n* = 12) Same injections with 5 mL PBS (*n* = 12) Experiment 2 (*n* = 3): Intratesticular injection 20 × 10^6^ BM-MSCs in PBS in 5 different points (1 mL in each point)	Experiment 1: No signs of inflammation. No differences on testicular volume, parenchyma echogenicity, testicular blood flow and serum testosterone levels between treated and control at 24 h and 28 h. Experiment 2: no physical alterations or changes in sperm parameters. Satisfactory fertility rate (83%; 5/6) after AI. The results suggest that MSC intra-testicular administration would be safe and would not affect testicular function.	No sperm parameters evaluated after BM-MSCs injection in experiment 1 Absence of control group in experiment 2 Small size of group in experiment 2

**Table 3:** In vivo studies using mesenchymal stem cells for integumentary system disorders. Umbilical cord-derived stem cells (UCB-MSCs), transforming growth factor beta (TGF-β), cyclooxygenase-2 (COX-2), intravenous (IV), HypoThermosol FRS (HTS-FRS), peripheral blood stem cells (PB-SCs), phosphate-buffered saline (PBS), amniotic fluid MSCs (AF-MSCs), platelet-rich plasma (PRP), carboxymethylcellulose (CMC), Wharton’s jelly MSCs (WJ-MSCs), bone marrow mesenchymal stem cells (BM-MSCs), adipose tissue MSCs (AT-MSCs).

Pathology	Study	Type MSCs	Experimental Design	Administration Regime	Outcome	Considerations
Traumatic wounds	Textor et al., 2017 [104]	Allogeneic UCB-MSCs	Six horses (3 mares, 3 geldings; 5–19 years) Induced model: 3 full-thickness cutaneous wounds surgically created on each distal forelimb Assessment and follow-up (6 weeks): Wound surface area Thermography Gene expression Histologic scoring	10–20 × 10^6^ normoxic UCB-MSCs, hypoxic UCB-MSCs or control in 1 mL NaCl 0.9% were injected into wound margins or topically applied embedded in an autologous fibrin gel, 1 day after wound creation	MSC administration by either delivery method was safe and improved histologic outcomes and reduced wound area over control MSC-injected wounds were consistently smaller than gel-treated or control wounds. Hypoxic pre-conditioned MSCs did not provide a substantial advantage	Treatments applied very early and in aseptically created wounds (proof of concept for application in clinical situation)
	Mund et al., 2020 [105]	Allogeneic UCB-MSCs	Two 7-year-old mares Determine adverse reactions after IV MSC administration and assess their engraftment potential into wounds Induced model: standardized cutaneous wounds surgically created on the left lateral third metacarpus (7 wounds) and left hemi-thorax (7 wounds)	1.02 × 10^8^ UCB-MSCs (fluorescently labelled) in 60 mL HTS-FRS via IV administration Control: 2 wounds were left to heal by second intention (in the same horses)	No clinically adverse effects (largest recorded dose of IV UCB-MSCs) UCB-MSCs preferentially engrafted into wounds during the acute and early remodeling phases.Results suggest no difference in homing potential between limb and thoracic wounds	Low number of animals Biopsy collection at the control sites created inflammation that may have influenced homing to the sequential control site (untreated control not included)
	Spaas et al., 2013 [106]	Autologous PB-SCs	4 horses with naturally occurring traumatic wounds unresponsive to conventional therapies for at least 3 months: Case 1 (11-year-old mare): dorsal surface of the metatarsal bone infected with *Clostridium* spp. Case 2 (16-year-old gelding): plantar surface of the metatarsal bone infected (presence of pus) Case 3 (26-year-old gelding): deep wound with bone exposition at the medial surface of the tibia Case 4 (26-year-old gelding): wound presenting non-neoplastic exuberant granulation tissue on the plantar surface of the metatarsal bone. Nodular proliferative lesions recurred after resection and tended to ulcerate.	5 × 10^5^ PB-SCs in 2 mL PBS were locally (intradermally) injected into 5–6 different locations at the wound’s edges and 1.25 × 10^5^ PB-SCs via IV administration	Granulation tissue began forming within 4 weeks of the PB-SC therapy in cases 1, 2 and 3. Crust formation was achieved within 2 months. In case 4, the granulation tissue could be easily removed without recurrence of the wound. 1 year: no wound recurrence or other adverse effects	Low number of cases of varying presentation with no control group, but unresponsive to previous treatments. Cells used not expanded and not fully characterized as MSCs. In cases with bacterial infection antibiotic, administration was continued.Outcome of each individual case is not deeply explained
Decubitus ulcers	Iacono et al., 2012 [110]	Allogeneic AF-MSCs and allogeneic PRP gel	One septic neonatal foal with severe ulcers in fetlocks, carpus and right stifle	Aloe gel every 48 h: Left hock and carpus 10 mL PRP gel: right carpus 5 × 10^6^ AF-MSCs + 10 mL PRP gel (twice a week for 2 weeks): right hock	None of the wounds treated developed exuberant scar tissue Healing was faster using AF-MSCs + PRP. The ulcer treated this way resulted in a linear scar, while the other lesions produced star scars 7 months: ulcer treated with aloe gel was not fully healed	One single case Different wound locations Concomitant disease Oral antibiotic therapy ongoing during ulcers healing
	Iacono et al., 2016 [111]	Allogeneic AF-MSCs	5 hospitalized neonatal foals (10–15 days old) with a total of 9 pressure sores on the carpus (4), fetlock (2), and hock (3). Sores were divided into group 1 (*n* = 6) and group 2 (*n* = 3)	Group 1: 5 × 10^5^ AF-MSCs in CMC gel applied twice a week for 2 weeks Group 2: formosulfathiazole ointment every 48 h	Sores treated with AF-MSCs in CMC gel healed quicker 30 days: no further treatments were needed	Low number of cases with no substance vehicle (CMC gel) treated control. Variable presentation of treated sores
	Lanci et al., 2019 [112]	Allogeneic WJ-MSCs	One 6-month-old filly hospitalized by the re-injury of a pressure wound on the left hock	5 × 10^6^ WJ-MSCs in a CMC gel were applied every 4 days for 4 times Four days after the last application, no further bandages were applied and the wound was daily cleaned and treated with hydrotherapy (cold tap water 10 min/day)	No side effects and fast wound regression No evident exuberant scar. The hair grew completely without changing color80% regression rate between 8 days and 39 days No relapse	One single case with no control
Laminitis	Morrison, 2011 [118]	Allogeneic UCB-MSCs	12 horses with naturally occurring chronic laminitis unresponsive to other treatments	20–25 × 10^6^ UCB-MSCs in NaCl 0.9% infused by regional perfusion (digital vein) every 3–4 weeks (3 infusions in total per affected foot)	83% of horses with positive evolution by the time of publication.	Routine treatments for laminitis continued Long-term success rates still need to be determined Only clinical follow-up Absence of control group No age or breed information stated
	Dryden et al., 2013 [114]	Allogeneic UCB-MSCs and autologous BM-MSCs	30 horses with naturally occurring chronic laminitis	20–30 × 10^6^ allogeneic UCB-MSCs in NaCl 0.9% infused by regional perfusion (digital vein) and subsequent injections with either 20–30 × 10^6^ autologous BM-MSCs or allogeneic UCB-MSCs (4 infusions in total at 1 month intervals)	21 patients (70%): successful outcome. Decreased radiologic distance between the bone and hoof wall Cases receiving MSCs < 30 days after the onset of laminitis: success rate was 100%. Cases receiving MSCs > 90 days after the onset of laminitis: success rate was 50%. Results suggest improved prognosis in cases treated early.	Routine treatments for laminitis continued Absence of control group Only clinical and radiologic follow-up Variation in therapeutic regime among cases. No age or breed information stated
	Angelone et al., 2017 [120]	Allogeneic AT-MSCs and autologous AT-MSCs	9 horses (5 mares, 4 geldings; 10–21 years) with severe naturally occurring laminitis unresponsive to conventional therapies	15 × 10^6^ allogeneic AT-MSCs in 15 mL autologous PRP infused by regional perfusion (digital vein) and subsequent injections with autologous AT-MSCs (3 infusions in total at 1 month intervals)	Clinical and radiologic signs improved All the animals returned to activity at six months from the first treatment. 12 months: 7 horses still performing activity 24 months: 2 animals relapsed and were euthanized 3 horses deceased (unrelated to laminitis)	Absence of control group Horses enrolled presented different laminitis stages. Only clinical and radiologic follow-up

**Table 4:** In vivo studies using mesenchymal stem cells in equine neurological disorders. Bone marrow mesenchymal stem cells (BM-MSCs), adipose tissue MSCs (AT-MSCs), cervical vertebral compressive myelopathy (CVCM), 99m technetium-hexamethyl-propylene-amine-oxyme (99mTc-HMPAO), atlanto-occipital (AO), lumbosacral (LS), recurrent laryngeal neuropathy (RLN), muscle-derived MSCs (M-MSCs).

Pathology	Study	Type MSCs	Experimental Design	Administration Regime	Outcome	Considerations
Peripheral nerve injury	Villagrán et al., 2016 [127]	Allogeneic BM-MSCs	Induced model: 3 healthy mares (9–13 years old) with surgically created 15-mm longitudinal incision over the *ramus communicans*	10 × 10^6^ BM-MSCs in 1 mL NaCl 0.9% or 1 mL NaCl 0.9% (control) instilled into the fascia surrounding the medial and lateral stumps Stumps of *ramus communicans* of each fore limb were harvested 45 days after treatment or control administration	No evidence of nerve regeneration No histological differences between MSC-treated and control nerve stumps No histological evidence of BM-MSCs or primitive cells (e.g., neural or Schwann-cell progenitors)	Small size Immediate treatment of aseptically created injury (proof of concept) Poor vasculature of the anatomical region may have influenced the outcome
CVCM	Barberini et al., 2018 [134]	Allogeneic AT-MSCs	Distribution and safety assessment in 6 healthy mares (6–21 years) and 3 diseased horses presenting moderate to severe neurological signs (presumedly CVCM) Assessment: Scintigraphy Detection of anti-AT-MSC alloantibody Necropsy (diseased horses)	Healthy horses: 100 × 10^6^ AT-MSCs in 5 mL NaCl 0.9% were injected either AO (*n* = 3) or LS (*n* = 3). One horse in each group received 99 mTc-HMPAO-labeled AT-MSCs. One additional horse was injected with free label as a control. Diseased horses: 100 × 10^6^ AT-MSCs via AO	Healthy horses: AO and LS intrathecal injection of relatively high doses of AT-MSCs was well tolerated AT-MSCs apparently distributed more efficiently through the subarachnoid space after AO injection (suggested as preferred route to deliver MSCs to the cervical area) Diseased horses: AT-MSCs not found at 15 days after injection at the site of injury (either did not have time to reach the lesion site or did not survive long enough) No horses developed detectable anti-AT-MSC alloantibodies.	Low number of horses per group. Tracking and control only in one horse
RLN	Sandersen et al., 2018 [137]	AutologousM-MSCs	5 healthy mares (ages 10–22) Evaluate the feasibility and safety of administering MSCs by a peri-neural injection to the left recurrent laryngeal nerve in healthy horses by using an electrical nerve stimulator	10 × 10^6^ M-MSCs in 1 mL cryopreservation medium directly administered into recurrent laryngeal nerve by a nerve stimulator-guided injection	Feasibility and safety of the procedure suggested by absence of functional changes upon endoscopic evaluation up to 28 days. No signs of adverse events in four out of the five mares up to 1 year after the injection	No control group M-MSCs administered immediately after thawing Composition of cryopreservation (administration) media not mentioned

## Data Availability

No new data were created or analyzed in this study. Data sharing is not applicable to this article.
